# Heat stress responses vary during floret development in European spring barley cultivars

**DOI:** 10.3389/fpls.2022.918730

**Published:** 2023-02-03

**Authors:** Cindy Callens, José Fernandez-Goméz, Matthew R. Tucker, Dabing Zhang, Zoe A. Wilson

**Affiliations:** ^1^ School of Biosciences, University of Nottingham, Loughborough, United Kingdom; ^2^ Waite Research Institute, School of Agriculture, Food and Wine, University of Adelaide, Urrbrae, SA, Australia; ^3^ Joint International Research Laboratory of Metabolic and Developmental Sciences, State Key Laboratory of Hybrid Rice, School of Life Sciences and Biotechnology, Shanghai Jiao Tong University, Shanghai, China

**Keywords:** barley, heat stress, floret development, meiosis, mitosis, pollen, male sterility, breeding

## Abstract

The Poaceae, or grasses, include many agriculturally important cereal crops such as rice (*Oryza sativa*), maize (*Zea mays*), barley (*Hordeum vulgare*) and bread wheat (*Triticum aestivum*). Barley is a widely grown cereal crop used for stock feed, malting and brewing. Abiotic stresses, particularly global warming, are the major causes of crop yield losses by affecting fertility and seed set. However, effects of heat stress on reproductive structures and fertility in barley have not been extensively investigated. In this study we examined three commercial European spring barley varieties under high temperature conditions to investigate the effects on floret development. Using a combination of fertility assays, X-ray micro computed tomography, 3-dimensional modelling, cytology and immunolabelling, we observed that male reproductive organs are severely impacted by increased temperature, while the female reproductive organs are less susceptible. Importantly, the timing of stress relative to reproductive development had a significant impact on fertility in a cultivar-dependent manner, this was most significant at pollen mitosis stage with fertility ranged from 31.6-56.0% depending on cultivar. This work provides insight into how heat stress, when applied during male pollen mother cell meiosis and pollen mitosis, affects barley fertility and seed set, and also describes complementary invasive and non-invasive techniques to investigate floret development. This information will be used to identify and study barley cultivars that are less susceptible to heat stress at specific stages of floral development.

## 1 Introduction

The world is facing an exponentially increasing population with associated increased demands for food, and all major climate models predict a higher average temperature globally with larger temperature fluctuations and more frequent heat waves (Christidis et al., 2014; IPCC, 2014). Abiotic stresses are, individually or in combination, one of the major reasons for crop yield loss. These stresses cause morphological, physiological, biochemical and molecular changes that impair plant development (Bita and Gerats, 2013). Lesk et al. (2016) showed that extreme heat significantly reduced national cereal production globally by 9-10%. Of particular concern is that three of the five most important cereal crops worldwide (i.e. wheat, maize and barley) exhibit the strongest negative yield impacts due to the changing climate (David and Christopher, 2007). Jacott and Boden (2020) recently reviewed the consequences of high ambient temperatures on wheat and barley, summarizing negative impacts on inflorescence development, the transition to flowering, and fertility. The increased frequency of heat stress over the last 40 years has clearly led to a greater frequency of yield anomalies in wheat (Zampieri et al., 2017), which accounts for 21% of food production. This proportion could be challenged with an increase in temperature in regions where temperatures are currently optimal (Ortiz et al., 2008; Delphine et al., 2014). Experiments in rice also showed that heat stress has an adverse effect on important yield components especially fertile spikelets and 1000 grain weight, decreasing yield significantly (Aghamolki et al., 2016). Similarly in spring barley, an increase in ambient temperature from 20°C/16°C (day/night) to 28°C/24°C reduced floret number and grains per spike (Hemming et al., 2012; Ejaz and von Korff, 2017). This serves as a warning for future global food security.

Reproductive organs are significantly more vulnerable to high temperatures than other plant organs. Planting of crops is typically timed to minimise high temperature exposure during the later reproductive stages, however the fluctuations and extremes of temperature that are now occurring mean that there is an increasing probability that peaks of high temperature will overlap with the flowering period (Teixeira et al., 2013; Kupke et al., 2022). It is proposed that “stay green” traits that enable photosynthesis to be maintained for longer, particularly under environmental stress, may enable yield losses due to abiotic stress to be minimized (Barnabas et al., 2008). Seed set requires many developmental steps to be successfully completed; for example, pollen must first be produced, viable pollen grains must be released and the pollen tubes must grow correctly to ensure functional signalling mechanisms with the style and the ovule. Concurrently, the ovule must develop within the ovary to produce and nourish the embryo sac, producing a female gamete and a suitable environment for the downstream events of seed development (Wilkinson et al., 2018). Once fertilization is complete, the embryo and endosperm must then develop normally to ensure successful seed fill (Maestri et al., 2002; Barnabas et al., 2008; Wilson and Zhang, 2009). Nevertheless, these developmental processes show variable sensitivity to heat stress (Jacott and Boden, 2020).

Prior to fertilisation, Saini et al. (1983) reported that one third of ovaries in wheat that experienced heat stress during meiosis exhibited abnormal development. Pollen, by contrast, exhibits much higher levels of abortion after heat stress at the same stage. Indeed, male reproductive development has been shown to be one of the most heat-sensitive stages in cereals (Saini et al., 1983; Stone and Basra, 2001; Prasad et al., 2008). At the pre-meiotic stage, high temperatures caused development of short anthers possessing no pollen grains in barley, while heat stress during meiosis resulted in pollen grains that possessed exine, but showed little starch accumulation (Sakata et al., 2000). Draeger and Moore (2017) also showed that exposure of wheat to high temperatures affected the progression of Pollen Mother Cell (PMC) meiosis. Disruption of synapsis is one of the most commonly reported meiotic failures under high temperatures and this can lead to unpaired univalents that segregate randomly or are lost (Bomblies et al., 2015). Elevated temperatures have also been shown to compromise mitosis 1 and 2 in both wheat and barley pollen (Saini et al., 1984; Barnabas et al., 2008). This may be due to an inability to synthesize all required Heat Shock Proteins (HSPs) necessary to survive during heat stress conditions (Cooper et al., 1984; Mascarenhas and Crone, 1996; Barnabas et al., 2008), and is consistent with causal involvement of HSPs in thermotolerance in non-cereal species (Hong and Vierling, 2000). Genotypes that express HSPs are better able to withstand heat stress as they minimise heat-induced protein aggregation and thus during the recovery period, facilitate their refolding (Nguyen et al., 1994; Feder and Hofmann, 1999; Farooq et al., 2011). In many cases, expression of HSPs is developmentally regulated and they are thus present prior to heat stress (Maestri et al., 2002).

One challenge of studying the response of florets to heat stress in cereals is determining when and where defects appear, how this differs between cultivars with different phenology and architecture, and how this might be examined in a non-destructive manner. In this study we analysed the effects of heat stress during floret development in three European spring barley varieties under controlled environmental conditions. One of the varieties, RGT Planet, represents a variety of considerable promise for high yield and malt quality in diverse conditions, including dryland environments in Australia (SeedForce, 2017). Our overall aim was to generate baseline data that might be used to develop further screening strategies and targets for reproductive heat tolerance during floret development in barley. We found that after heat stress, anthers from all varieties showed abnormal development, whilst ovules were less severely affected. The three varieties tested showed differences in sterility, confirming their varying tolerance to heat stress. RGT Planet exhibited excellent yield under control conditions, but suffered significantly after heat stress. In contrast to Moonshine and RGT Planet, Optic was the most severely affected by heat stress at pollen mitosis during floret development.

## 2 Materials and methods

### 2.1 Plant materials, growth and heat stress conditions

Seeds from three different spring barley varieties (*Hordeum vulgare*; Optic (derived from the cross Chad × (Corniche × Force), RAGT Moonshine and RGT Planet) were provided by the Wilson lab (University of Nottingham) and were sown in John Innes Potting Compost No.3 in 13cm diameter pots for germination and early plant establishment. All three are elite lines that have been on the AHDB Recommended List, although RAGT Moonshine and Optic were removed from the AHDB Recommended List in 2016 (AHDB, 2016). Optic is a variety created by Syngenta, while RAGT Moonshine and RGT Planet are varieties created by RAGT Seeds. RGT Planet is a relatively new variety that has been shown to be one of the highest yielding spring varieties on the AHDB Recommended List that is fully approved for brewing use (AHDB, 2017). All three are two-row varieties and are primarily grown in the United Kingdom, although RGT Planet is also popular in Australia. Temperatures of 15-20°C are considered optimal for growth and development of spring barley varieties (Kruszka et al., 2014).

After sowing, pots were placed in a controlled environment growth chamber with a continuous temperature of ~17°C and 16/8 -hour light/dark photoperiod and 80% relative humidity. After one week, the plants were transferred to CSNG (General Container Nursery Stock compost, Levington Advance) compost in 13cm diameter pots. Heat stress was applied to two stages of reproductive development (Pollen Mother Cell meiosis and pollen mitosis, **Supplementary Figure S1**). Staging of reproductive development was conducted according to the non-destructive staging described in Gómez and Wilson (2012), to ensure that all samples were at the appropriate and equivalent developmental stage. Pollen Mother Cell Meiosis corresponded to spike stage 4, spike one-quarter within previous last sheath; pollen mitosis corresponded to spike stage 13, spike half out (Gómez and Wilson, 2012). Stressed plants were compared to plants grown in control conditions to ensure there was a matching of the corresponding developmental stages. Male as well as female reproductive development were simultaneously targeted because the onset of the PMCs in anthers coincides approximately with meiosis in the megaspore mother cell (Saini et al., 1983).

Twelve plants of each variety were used for each condition: 1) Pollen Mother Cell (PMC) meiosis heat stress, 2) Pollen mitosis heat stress and 3) control. The periods of heat stress were carried out in a controlled environment growth room with a day/night temperature of 30/25°C, 80% relative humidity and a 16/8 -hour light/dark photoperiod to mimic rapid onset heat stress conditions. The temperatures were chosen to mimic heat waves occurring more frequently in Europe. Tillers were selected at the beginning of meiosis or mitosis, and were tagged for analysis before being submitted to heat stress. All pots were monitored daily and watered to avoid concurrent drought stress. Plants undergoing the PMC meiosis heat treatment were submitted to heat stress for two days, while plants undergoing the Pollen mitosis heat treatment remained in the heat stress conditions for five days to ensure completion of pollen mitosis I and II, before being transferred back to control conditions. Control plants were maintained at a constant 17°C, 80% relative humidity with a 16/8 -hour light/dark photoperiod.

### 2.2 Pollen viability assay

Pollen viability in control and heat stressed plants was assessed by analysis of starch levels in pollen grains using 0.2% (w/v) potassium iodide and 1% (w/v) iodine to determine their ability to germinate and fertilize (Chang et al., 2014). Normal mature pollen grains that contain starch granules stain black, whilst immature pollen grains or deformed pollen grains appear orange, or red. Pollen viability images were taken with a Nikon Eclipse 50i microscope and a Nikon DS-Fi1 camera.

### 2.3 Thin sectioning and cell wall immunolabelling

Florets were harvested from the tillers tagged for heat stress during pollen meiosis immediately before anthesis, fixed in 4% (w/v) paraformaldehyde in phosphate-buffered saline (PBS) with 0.1% (v/v) Triton X-100 and 0.1% (v/v) Tween 20 and embedded in paraffin. Floret sections (8μm) were mounted on slides at 42°C, the paraffin was removed from the sections using 100% Histoclear (v/v) and tissue was rehydrated by using an ethanol series (100%, 90%, 70% and 30% (v/v)) and water. Slides were washed with 1xPBS and treated subsequently with glycine to inactivate residual aldehyde groups. They were then washed with Incubation buffer (1% (w/v) bovine serum albumin (BSA)) in 1xPBS (Burton et al., 2011). Primary antibody was added to the sections and incubated in a humidity chamber for 1h: BG1 murine monoclonal antibodies raised against barley (1,3;1,4)-β-d-glucan (diluted 1:50; Biosupplies Australia, Parkville, Vic., Australia) and LM19 monoclonal antibodies raised against homogalacturonan (Meikle et al., 1994; Verhertbruggen et al., 2009; Burton et al., 2011). After three washes with Incubation buffer the slides were dried and the secondary antibodies added. Goat anti-mouse Alexa Fluor^®^ 488 IgG (H+L) (diluted 1:200, Invitrogen, Australia) was used for BG1 and Dylight 550 Goat anti-rat IgM (diluted 1:200, Invitrogen, Australia) was used for LM19. The slides were incubated for 2h and then washed with Incubation buffer. 0.1% (w/v) Calcofluor white was added and washed off before imaging with the Zeiss Axio Imager 2 as described in Aditya et al. (2015).

### 2.4 3D X-ray computed tomography imaging

The Phoenix Nanotom S, a nanofocus Computed Tomography (CT) system was used to scan barley florets of the three varieties at the Hounsfield Facility in Nottingham as previously described (Tracy et al. (2017). Control and heat stressed florets (heat stress applied during PMC meiosis) were scanned and the scans were segmented and analysed using VG Studio Max 2.2.

### 2.5 Reproductive organ phenotyping

Five different, randomly chosen florets were harvested immediately before anthesis after the heat treatments and in control conditions from all varieties and dissected to observe the morphology of the anthers. Images were taken with a Zeiss Stemi SV6 microscope and an Axiocam ERc Rev. 2.0 camera.

### 2.6 Evaluation of sterility

All tillers from control and heat stressed plants were harvested and the numbers of seeds per spike counted (**Table 1**) to determine the levels of fertility. For every spike the total number of spikelets and the number of spikelets that developed into seeds were counted. The reduction of fertility due to heat exposure was expressed as the percentage of sterility per tiller, by dividing the number of seeds formed by the total number of spikelets and multiplying by 100. Tagged heads from the plants were harvested and the percentage of sterility was determined on a tiller-by-tiller basis for each heat treatment and control conditions using a Kruskal-Wallis (one-way ANOVA) test to compare between treatments and varieties.

**Table 1:** Average number of tillers per plant and seeds per spike, and number of tagged tillers for the three different varieties in control conditions, after PMC meiosis heat treatment or after pollen mitosis heat treatment ± standard deviation.

Variety	Treatment	Average number of tillers/plant	Number of tagged tillers	Average number of seeds/spike	Percentage fertility for each variety per spike compared to control
Optic	Control	7 ± 1.8		19 ± 2.8	100
	Meiosis	6 ± 0.7	8	14 ± 4.6	73.7
	Mitosis	5 ± 0.9	12	6 ± 3.8*	31.6
Moonshine	Control	7 ± 1		25 ± 2.0	100
	Meiosis	6 ± 0.7	5	16 ± 3.4*	64.0
	Mitosis	6 ± 1.1	16	14 ± 2.4*	56.0
RGT Planet	Control	6 ± 1.2		28 ± 2.8	100
	Meiosis	6 ± 1.6	12	12 ± 3.4*	42.9
	Mitosis	6 ± 1.3	13	13 ± 4.1*	46.4

Asterisks indicate a p-value <0.05 compared to the control.

### 2.7 Phylogenetic analysis of heat shock proteins in barley

To determine orthologs of *AtHSP17.8* (AT1G07400.1) and *AtHSP70* (AT3G12580.1) a nucleotide BLAST search was carried out in NCBI, rice database and IPK Barlex database. Orthologs were identified for rice (*OsHSP17.8*, LOC_Os03g16030.1 and *OsHSP70*, LOC_Os11g47760.1) and barley (*HvHSP17.8*, AK368988.1 and *HvHSP70*, HORVU5Hr1G021300.3 and HORVU6Hr1G081460) and verified by multiple alignment through phylogenetic tree analysis (**Supplementary Figure S2**). The alignment was constructed using the Geneious alignment method and the Blosum62 cost matrix in Geneious (version 8.0 by Biomatters, available from http://www.geneious.com). These proteins were chosen to give an indication of differences between varieties in the response of heat shock proteins to heat stress. Due to the high similarity between the proteins in the cytosol subgroup of the HSP70 family, we focussed only on the closest orthologues in barley.

### 2.8 Quantitative reverse transcription (qRT) PCR

RNA was extracted with the RNeasy^®^ Plant Mini Kit (Qiagen, UK) from at least five florets and pooled together. cDNA was synthesised from total RNA using the SuperScript™ III reverse transcriptase (ThermoFisher Scientific, UK). qRT-PCR was performed with primers for *HvHSP17.8* (HvHSP17.8 Fw 5’GAGGTGGAGGACGGCAACA3’ and HvHSP17.8 Rv 5’GATGGACTTGATCTCGGGTTT3’) and *HvHSP70* (HvHSP70 Fw 5’ATCCTGAACGTGTCTGCTGA3’ and HvHSP70 Rv 5’TGGTGTTGCGCATGTTGTAA 3’) on three technical replicates and analysed using the LightCycler^®^ 480 (Roche Life Science, UK). Expression values relative to the barley α-Tubulin gene (HvTubF 5’AGTGTCCTGTCCACCCACTC3’ and HvTubR 5’AGCATGAAGTGGATCCTTGG3’) were calculated.

## 3 Results

### 3.1 Anther morphology in barley varieties after heat stress

**Figure 1:**
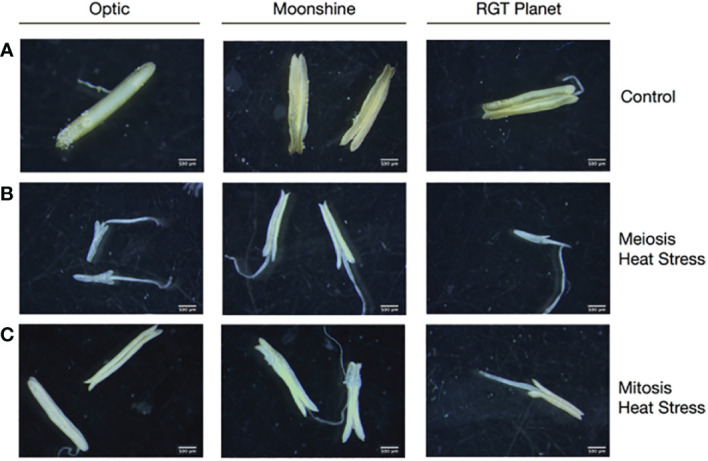
Anther development in barley cultivars Optic, Moonshine and RGT Planet. Representative anthers dissected from a minimum of three florets from Control **(A)** and heat stressed florets, **(B)** Heat Stress during Pollen Mother Cell meiosis and **(C)** Pollen Mitosis show differences in anther development under the different conditions.

**Figure 2:**
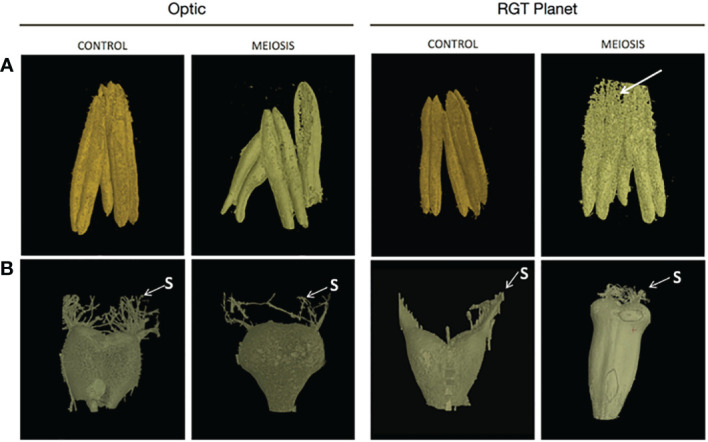
X-ray CT images of **(A)** anther and **(B)** carpel development from a representative floret from control and heat stress conditions for barley cultivars Optic and Planet. X-ray images of the anthers and carpels obtained by X-ray CT were compared for control environment and heat stress environment (heat stress during meiosis of pollen development) immediately before anthesis. Arrow indicates the broken septum in RGT Planet heat stressed anther.

Anthers were dissected from at least three randomly selected florets for the different varieties (Optic, Moonshine and RGT Planet) that had been exposed to heat treatment at PMC meiosis and Pollen mitosis, and compared to those from the control conditions. Staging of anther development was conducted according to the non-destructive reproductive staging approach of Gómez and Wilson (2012), this ensured that heat stress was delivered at the appropriate developmental stage and that comparisons between samples were consistent based upon developmental stage. All of the heat-treated lines showed effects on anther morphology, with alterations in anther shape and a change in colour from yellow to white (**Figure 1**). In all three varieties, the effect on anther morphology was more severe after heat stress during PMC meiosis compared to Pollen mitosis, but both treatments showed impacts on anther shape and colour. In particular, Optic and RGT Planet showed a severe change in PMC meiosis heat stressed anthers (**Figure 1**). Anther morphology was investigated further using 3D images obtained from X-ray micro-CT scans of a representative floret from Optic and RGT Planet (**Figure 2**) and RAGT Moonshine (**Supplementary Figure S3**). Anther volume varied significantly; control non-stressed anthers were approximately 0.9mm^3^ (4.33mm length, 1.98mm width, 1.43mm depth) in Optic and 1.1 mm3 (4.49 mm length. 2.83 mm width, 1.37 mm depth) in RGT Planet. Whereas the PMC meiosis heat stressed anthers were smaller: 0.29 mm^3^ (3.91 mm length, 3.01 mm width, 1.82 mm depth) in Optic and 0.71 mm^3^ (3.1 mm length, 2.35 mm width, 1.01 mm depth) in RGT Planet (**Figure 2**). The septum in the heat-stressed anthers could be observed as broken in the micro-CT scans, while this was not the case for the control anthers (**Figure 2A**).

**Figure 3:**
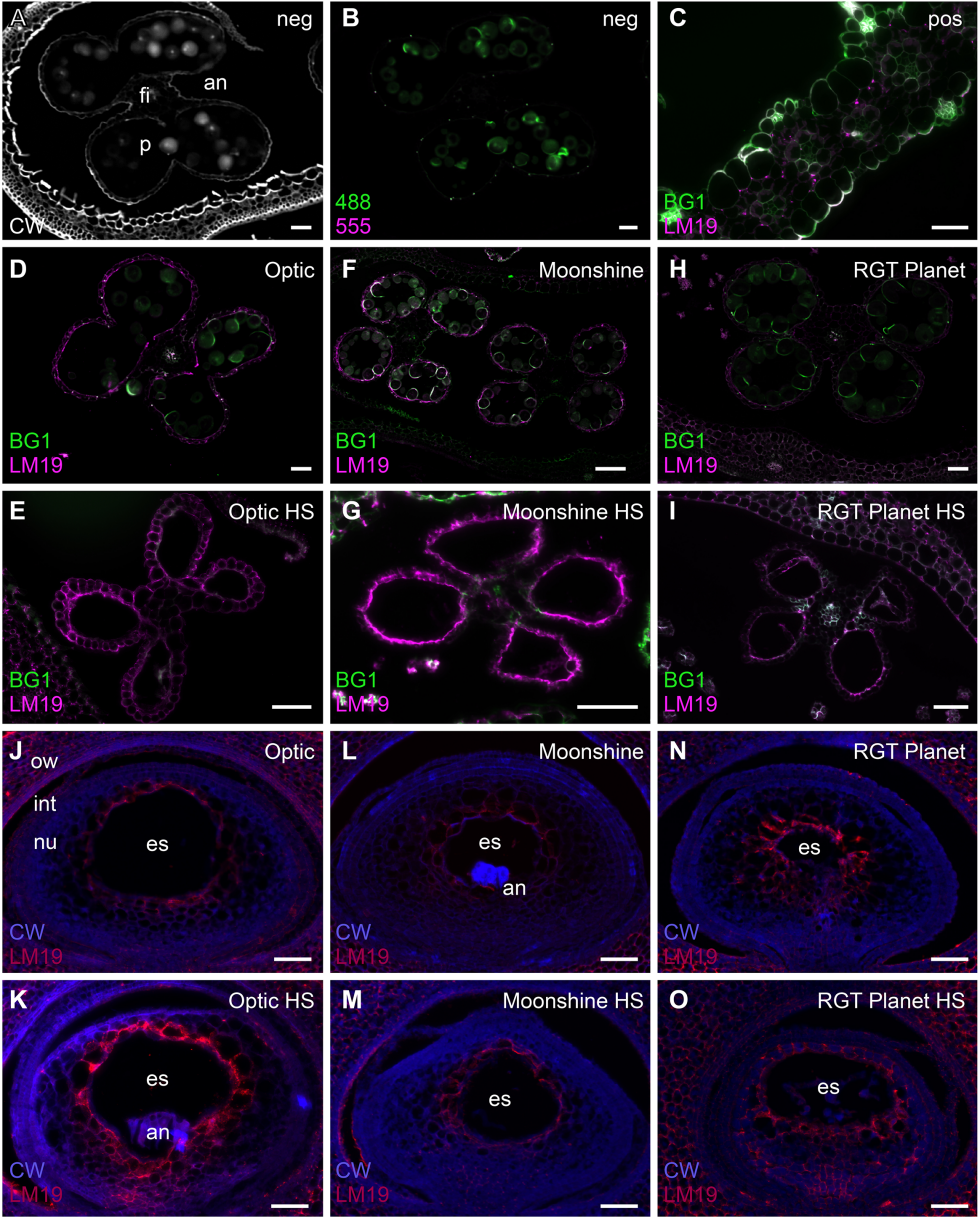
Immunolabelling of cell wall components in anthers and ovules in Optic, Moonshine and Planet. **(A)** Calcofluor white staining (CW, white colour) of a control anther (an). The filament (fi) and pollen (p) are indicated. **(B)** The same anther shown in A, showing autofluorescence (green) in the Alexafluor-488 channel. **(C)** A barley leaf sample showing positive labelling of (1,3;1,4)-β-glucan (BG1, green) and de-esterified pectin (LM19, magenta). **(D–O)** Comparison of control and heat stressed anthers and ovules in barley florets of the three varieties. Merged images of the antibody labelling patterns are shown. **(D)** Optic control anther. **(E)** Optic heat-stressed anther. **(F)** Moonshine control anther. **(G)** Moonshine heat-stressed anther. **(H)** Planet control anther. **(I)** Planet heat-stressed anther. **(J)** Optic control ovule. The ovary wall (ow), integuments (int), nucellus (nu) and embryo sac (es) are indicated. **(K)** Optic heat-stressed ovule. In this ovule the antipodals (an) are also evident. **(L)** Moonshine control ovule. **(M)** Moonshine heat-stressed ovule. **(N)** Planet control ovule. **(O)** Planet heat-stressed ovule. Bar = 50µm in all images. Images are representatives from at least 3 different florets per variety/heat stress treatment.

Immunolabelling was subsequently used to observe sections of the anthers and highlight cell wall components such as 1,3;1,4-β-glucan, de-esterified homogalacturonan and cellulose to determine if the cell wall composition was impacted as a consequence of the heat stress (**Figure 3**; **Supplementary Figures S4**-6). Calcofluor white stained β-glycan polysaccharides in the outer layers of the anther, the filament vascular tissue and the pollen grains (**Figure 3A**). Some autofluorescence was detected in the pollen grains, particularly in the 1,3;1,4-β-glucan channel (**Figure 3B**), but positive controls confirmed 1,3;1,4-β-glucan and HG labelling (**Figure 3C**). The results clearly indicated that the meiosis heat-stressed anthers of all the lines were significantly impacted by the stress and labelling of HG in the anther walls appeared to be more intense compared to control anthers (**Figures 3E, G, I**). In Optic (**Figures 3D, E**), pollen was visible in the control anthers, but not in the PMC meiosis heat-stressed anthers. The epidermal cells of the anther wall were intact, but the endothecium had degraded. Both tissue layers were intact and visible in the Optic control anthers (**Supplementary Figure S4**). In Moonshine (**Figures 3F, G**), no pollen was visible in the PMC meiosis heat-stressed anthers despite it being observed in the micro-CT scanned images (**Supplementary Figure S3**). The epidermis was also intact, but the endothecium had degraded in the heat stressed anthers (**Supplementary Figure S5**). Similar to the other two varieties, while pollen grains were visible in the control anthers, none were visible in the heat stressed anthers in RGT Planet (**Figures 3H, I**). The anther wall in the heat stressed anthers seemed to have collapsed, which agrees with the observations from the micro-CT scan (**Figure 2**; **Supplementary Figure S5**).

### 3.2 Carpel morphology in barley varieties after heat stress

The 3D images of the carpels from Optic showed no significant changes in morphology between the PMC meiosis heat-stressed and control florets, but there was a difference in size (**Figure 2**; **Supplementary Figure S4**). The control carpel measured 1.31mm^3^ (2.71mm length, 2.35mm width, 1.45mm depth), which is smaller than the heat stressed carpel, which measured 2.58mm^3^ (2.86 length, 2.77mm width, 1.34 depth). The X-ray imaging also showed a significantly enlarged carpel in the RGT Planet heat-stressed floret with a volume of 4.46mm^3^ (5.37mm length, 2.51mm width, 1.57mm depth) compared to the control which had a volume of 1.37mm^3^ (2.42mm length, 2.22mm width, 1.13mm depth) (**Figure 2**).

The immunolabelling of ovule sections in Optic only showed slight differences between control and heat stressed florets (**Figures 3J, K**): the embryo sac was present in all cases, but there seemed to be less cell layers between the embryo sac and the integument, and nucellus cells appeared larger. This could be due to regional nucellar degeneration and/or faster development of heat-stressed ovules. Similarly, embryo sac morphology in the heat-stressed ovules of Moonshine did not show any significant difference compared to control ovules (**Figures 3L, M**). The ovules in the heat-stressed florets of RGT Planet seemed to be further developed than the controls (**Figures 3N, O**), but no obvious irregularities in the morphology of the embryo sacs could be identified.

### 3.3 Male fertility after heat stress

PMC meiosis and pollen mitosis stages are known to be vulnerable to heat stress during pollen development, therefore pollen viability was assessed using potassium iodide/iodine solution in anthers from all three varieties with and without heat treatment. Using this method, viable pollen stains black due to starch deposition whilst non-viable pollen stains orange-red. Despite the lack of pollen identified in thin sectioning, pollen was still present in anthers from some florets after heat stress at PMC meiosis and pollen mitosis, but there was a lack of starch staining indicating a high level of sterility (**Supplementary Figure S1B**). Pollen could be observed in the Optic and RGT Planet anthers, after both PMC meiosis and pollen mitosis heat stress, but was far less abundant than in the control plants, and poor staining suggested that little if any was viable. Moonshine was even more strongly affected with no pollen present after the PMC meiosis heat stress. After heat stress during pollen mitosis, viable pollen was observed in Moonshine but at a lower amount than in the control anthers. The impact of heat stress varied depending on developmental stage and cultivar, with increased sterility seen for heat stress during the pollen mitosis stage; with 26-57% sterility observed for heat stress at the PMC meiosis stage, whilst 44-68% sterility was seen when heat stress occurred at pollen mitosis stage (**Table 1**).

### 3.4 Seed set after heat stress

The number of tillers and seeds per spike was assessed collectively for the three varieties after both treatments and in control conditions (**Table 1**). In Optic, there was no significant difference between the average number of seeds per spike after heat stress during PMC meiosis (~14 seeds) and the control (~19 seeds). However, there was a significant reduction of average seed number per spike after pollen mitosis heat stress compared to the control, from 19 to 6 seeds per spike. In Moonshine there was a significant decrease in seed set after both PMC meiosis and pollen mitosis heat treatments, from 25 in the control conditions to 16 and 14 respectively, after stress. The same could be observed in RGT Planet where a significant decrease was identified after both heat treatments (**Table 1**). In control conditions, there was an average of 28 seeds per spike, while after PMC meiosis heat stress this was reduced to 12 and after pollen mitosis heat stress to 13. These results contrasted with the lack of pollen and severe defects in pollen viability described above. However, it likely reflects variation in the response of different florets on each spike (depending on position) and different tillers depending on their developmental stage

**Figure 4:**
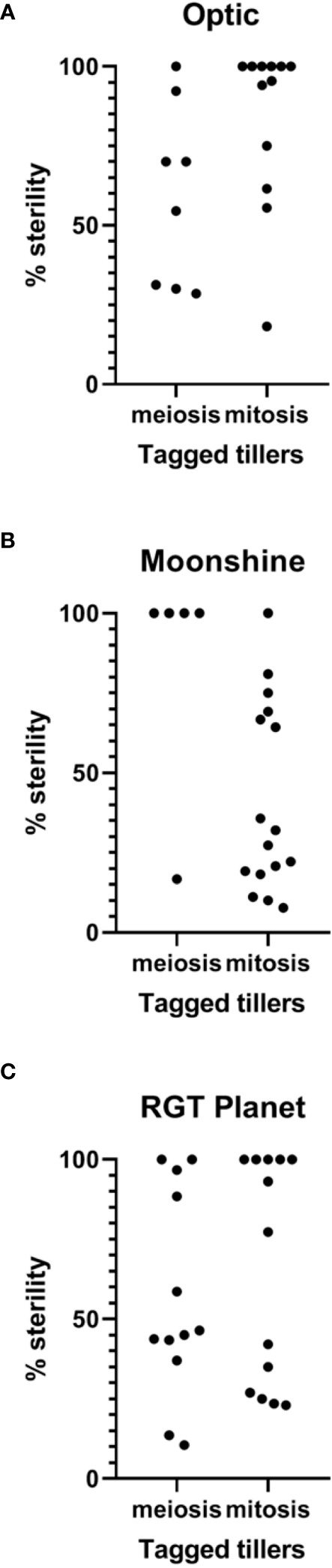
Sterility in heat stressed and control conditions in three European barley varieties. Percentage of sterility was calculated for all tillers for all three varieties after PMC meiosis and pollen mitosis heat treatments for **(A)** Optic **(B)** Moonshine and **(C)** RGT Planet. Kruskal-Wallis test was used to determine significance of difference between varieties and between treatments. All analysis was based upon based upon at least three biological replicates.

**Figure 5:**
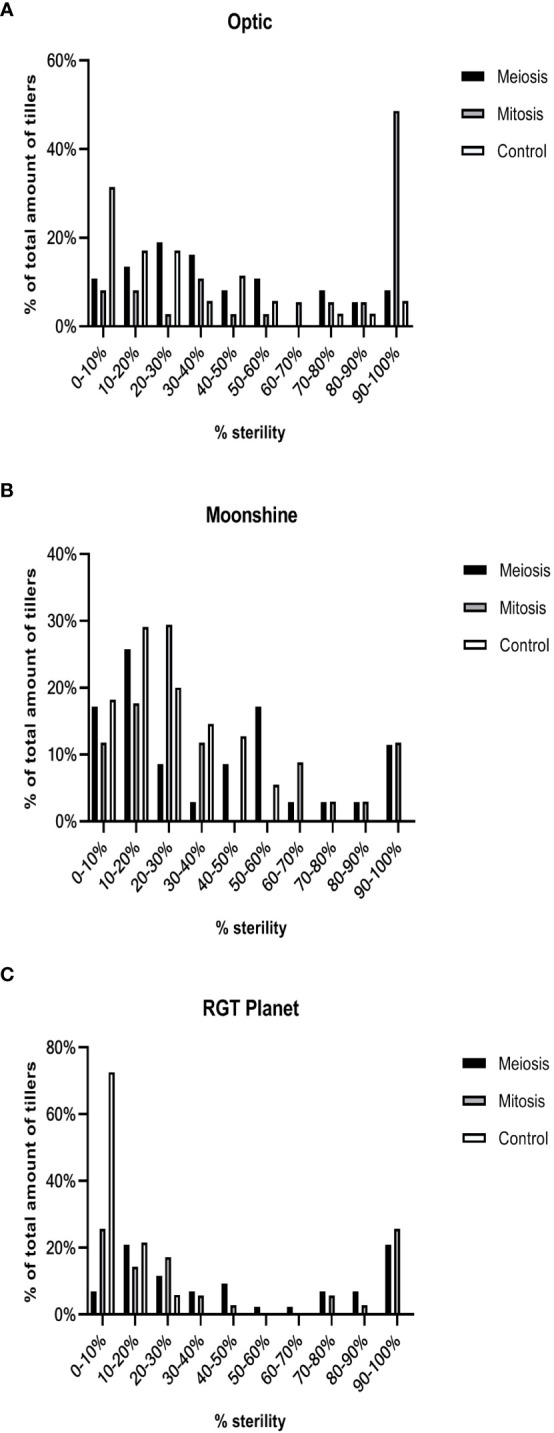
Frequency distribution of sterility in heat stressed and control conditions in three European barley varieties. The percentage sterility (florets that did not produce seed) was calculated for all three varieties in control conditions and after meiosis or mitosis I & II heat treatment for all pots **(A–C)**.

To partially address this variability, all tagged heads from the plants were harvested and the percentage of sterility was determined on a tiller-by-tiller basis for each heat treatment and in control conditions for all varieties. A Kruskal-Wallis test was used to compare between treatments and between varieties (**Figures 4**, **5**). Even though some spikes showed significant levels of sterility in Optic, consistent with the overall analysis, there was no significant difference in the percentage of sterility between PMC meiosis heat treatment and control conditions. There was however a difference between pollen mitosis heat treatment and control conditions, showing a significant increase in sterility (**Figure 4A**). In Moonshine there was no significant difference between both heat treatments and the control conditions, despite the majority of PMC meiosis heat treated tillers exhibiting 100% sterility. In this case, overall significance was likely confounded by a small sample size and a single tiller that showed relatively normal fertility (**Figure 4B**). In contrast, Moonshine did appear to be particularly tolerant to heat treatment at the pollen mitosis stage. In RGT Planet the sterility was significantly different between both heat treatments and the control (**Figure 4C**). Comparison between varieties showed that there was no significant difference after PMC meiosis heat stress (**Figure 4B**). After pollen mitosis heat treatment there was no significant difference between Moonshine and RGT Planet, but there was a significant difference between Moonshine or RGT Planet and Optic, the latter having increased sterility compared to the other two varieties. In control conditions Moonshine and Optic showed similar yields, while RGT Planet had a significantly higher yield.

### 3.5 Genes encoding heat stress proteins, HvHSP17.8 and HvHSP70, are induced after heat stress applied during mitosis

**Figure 6:**
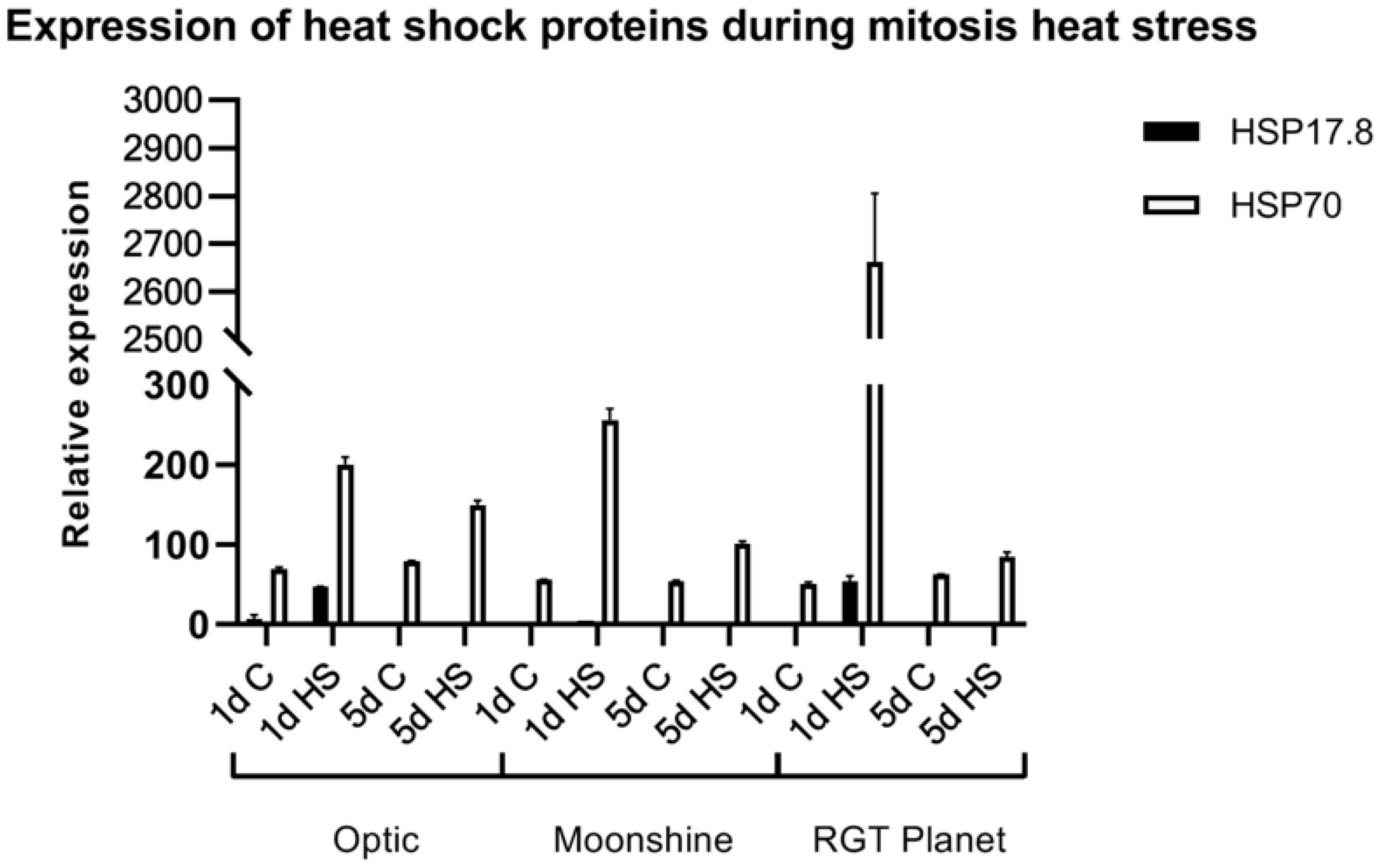
Expression of Heat Stress Proteins, HvHSP17.8 and HvHSP70, in florets of three European barley varieties, Optic, Moonshine and RGT Planet, during heat stress at the pollen mitosis I & II stages. C: control no heat stress; 1 and 5 days heat stress. Error bars indicate standard error, based upon at least three biological replicates.

To determine the impact of heat stress on gene expression, two well characterized Arabidopsis heat stress proteins were investigated in barley; *HvHSP17.8* is a small heat shock protein (sHSP) involved in protein stability, while *HvHSP70* is a chaperone involved in protein folding (Hartl, 1996; Wang et al., 2004). The putative barley orthologues of the two *Arabidopsis* heat stress genes [*HvHSP17.8* and *HvHSP70*, identified by BLAST and multiple alignment analysis (**Supplementary Figure S2**)] were analysed in florets as exemplars of gene expression changes after heat stress. The relative expression of *HvHSP17.8* and *HvHSP70* was examined on the first and last day (day 5) of pollen mitosis heat stress for all three varieties and control plants (**Figure 6**). *HvHSP17.8* was significantly upregulated after one day of heat stress in Optic and RGT Planet, but not in Moonshine. Expression of *HvHSP17.8* had returned to the minimal background level after five days of heat stress. *HvHSP70* was upregulated in all three varieties after one day of heat stress, most significantly in RGT Planet. Although *HvHSP70* expression decreased after five days of heat stress, it was still upregulated in all three cultivars compared to the controls.

## 4 Discussion

Three elite barley varieties were assessed for their response to heat stress during floret development. In all cases, plants that experienced heat stress at two key stages (PMC meiosis and pollen mitosis) showed severe morphological deficiencies in anther development, and these are likely to be a leading cause of sterility due to high temperature stress. A short period of heat stress during early floret development resulted in significantly smaller and deformed anthers. Although the anthers were harvested at the same time from heat stressed and control plants, the septa in the heat stressed anthers had already broken, suggesting a more rapid developmental progression and/or a change in the physical properties of the anther walls. It has been suggested that thick locule walls and well-developed cavities in the septa may be responsible for heat tolerance in rice (Matsui et al., 2001). The cavities are hypothesised to enable easy rupture of the septa in response to the swelling of the pollen grains, while the thick locule walls promote the swelling of the pollen grains by retaining water in the locules (Matsui et al., 2001). In the anthers examined here, the locule walls and cavities seemed less developed in the heat stressed plants, which might be another reason why the pollen grains did not develop correctly.

Saini et al. (1984) described two types of anther defects after heat stress in wheat. Type 1 typically had premature tapetal degeneration which resulted in periplasmodial invasion of the locule at meiosis and ultimately led to sterility. Degeneration of the outer layers of the anther wall was also observed. The second type was characterised by microspores that completed pollen grain mitosis I (PGM1), but a proportion of which became disoriented from the tapetum and developed no further. The breakage of the septa identified in heat-stressed barley might also be attributed to the fragility of the anther walls, as we observed some degeneration of anther wall layers in the immunolabelled images from RGT Planet.

In addition to defects in anther morphology, pollen development was severely affected by heat stress during both PMC meiosis and pollen mitosis, with decreased viability, pollen count, and accumulation of starch. However, as shown by the X-ray micro CT images of Moonshine florets after PMC meiosis heat stress, and by the presence of viable pollen in Optic after meiosis heat stress, pollen is still formed in some heat-stressed florets and, based on KI staining and seed counts, some may be viable. The absence of pollen detected in anthers during immunolabelling may be due to early anther rupture and loss of pollen during fixation and embedding. Alternatively, many florets may lack pollen entirely, and pollen is only released from some florets that avoid stress based on their position within the spike and their stage of development, which varies between the central and terminal regions. This highlights the importance of optimising non-destructive X-ray CT to assess anther development along the spike *in vivo* after heat stress.

Despite apparent contradictions between the immunolabelling experiments, KI staining assays, and CT data in regard to pollen grain number and viability after heat stress, seed set was used as a functional output of floret fertility. This varied between the treatments and cultivars. In Moonshine, overall seed set was significantly reduced across many tillers after heat treatment at PMC meiosis or pollen mitosis, but analysis of individual spikes suggested that Moonshine was more resilient against heat stress at the pollen mitosis stage. Optic, on the other hand, showed no significant response to heat stress at PMC meiosis, but was strongly sensitive at pollen mitosis. In contrast, RGT Planet was sensitive to heat stress at both PMC meiosis and mitosis stages of pollen development. These results potentially reveal different temporal sensitivities to heat stress in three elite barley cultivars.

The reduction in floret fertility and seed number may result from multiple defects in the pollen after heat stress. This includes reduced starch accumulation, which was observed in Optic pollen after heat stress at pollen mitosis. It has been suggested that stresses such as water stress can inhibit starch deposition in rice and wheat pollen, either by decreasing the availability of assimilates or by impairing the activities of enzymes involved in starch biosynthesis (Sheoran and Saini, 1996; Ji et al., 2010). Pressman et al. (2002) found that continuous high temperatures prevented the transient increase in starch concentration in tomato pollen grains which led to decreases in the concentrations of soluble sugars in the anther walls and the pollen. They concluded that this might contribute to decreased pollen viability in tomato after heat stress. This has been confirmed in other species, such as sorghum (Jain et al., 2007), and has been supported by evidence that barley grains from heat stressed plants accumulated less starch than grains from control plants due to reduced conversion of sucrose to starch (Wallwork et al., 1998). Heat stress has been reported to have a negative effect on the activities of enzymes involved in the sucrose-to-starch metabolism in cereals which might explain the reduction in starch content (Duke and Doehlert, 1996; Wilhelm et al., 1999; Hurkman et al., 2003).

The female reproductive organs did not show any significant differences in embryo sac phenotypes after heat stress. However, the carpels were bigger, possibly as a result of swelling of the unfertilized ovaries similar to that observed in wheat (Okada et al., 2018). Moreover, the carpels and ovules appeared to have developed faster than the controls. This suggests that in the three varieties under examination, the female reproductive organs are not as severely impacted by heat stress as anthers at the PMC meiotic and pollen mitotic stages of development, but advance more quickly through development. The broken septa in the anthers in the heat stressed plants also indicate that plants exposed to short pulse of heat stress around PMC meiosis might respond by speeding up reproductive organ development. A similar phenomenon was previously reported where heat stress hastened spike development and reduced spike number, thus impacting the number of grains per spike (Halse and Weir, 1974; Saini and Aspinall, 1982; Johnson and Kanemasu, 1983).

Several studies indicate that both pre- and post-fertilisation stages of ovary development are sensitive to stress, but this varies depending on the species and genotype (Sun et al., 2004; Zinn et al., 2010; Bac-Molenaar et al., 2015; Onyemaobi et al., 2017). In wheat, plants exposed to severe heat stress at the start of meiosis experienced disrupted nucellus and integument development, and complete ovule abortion at a frequency of 30% (Saini et al., 1983). In contrast, little is known about the effects of abiotic stress on cellular morphology and the regulatory network of the carpel and ovule in barley. The nucellar cells in all three barley cultivars examined here, particularly Optic, appeared to be disorganised and enlarged after heat stress relative to controls. However, in all cases, similar immunolabelling patterns for de-esterified homogalacturonan were observed around what appeared to be an intact embryo sac. Barley only has one ovule per floret and therefore needs to safeguard its one chance of survival. The multi-layered nucellus might be one of the reasons the developmental program of the ovule is more robust than that of the anther (Wilkinson et al., 2018).

The expression of two heat shock protein genes, *HvHSP17.8* and *HvHSP70*, were upregulated after heat stress. *HvHSP17.8* is a small heat shock protein (sHSP) that assists in preventing aggregation and stabilizing proteins while *HvHSP70* is a chaperone that assists in protein folding processes (Hartl, 1996; Wang et al., 2004). Both genes have been shown to be upregulated under stress conditions and to confer heat stress tolerance (Sung et al., 2001; Guo et al., 2009; Montero-Barrientos et al., 2010). *HvHSP70* was upregulated in all three varieties after one day and also after five days of heat stress (**Figure 6**). *HvHSP70* expression showed a peak after one day of pollen mitosis heat stress in RGT Planet, with much higher expression than in the other two varieties. Optic showed the least amount of upregulation of *HvHSP70*, which correlated with the elevated level of sterility in this cultivar after pollen mitosis heat stress. In contrast, *HvHSP17.8* was upregulated in Optic and RGT Planet after one day of heat stress at the pollen mitosis stage, but levels were indistinguishable from controls after five days of heat stress. This might indicate that *HvHSP17.8* is involved in the early response to heat stress. Curiously, a significant increase in *HvHSP17.8* was not observed in Moonshine despite this cultivar appearing to be less susceptible to heat-stress at the pollen mitosis stage. One possibility is that the timing of *HvHSP17.8* induction differs in Moonshine; alternatively, other HSPs may be induced and contribute to the higher levels of stress tolerance observed in this cultivar.

## 5 Conclusions

A short period of heat stress during the reproductive phase in barley is detrimental for the development of the male reproductive organs, but less so for the female reproductive organs. Results indicate that after heat stress, floret development was generally hastened, which might reflect an overall stress response from the plant to ensure seed set, albeit with a smaller amount of seeds. Prolonged heat stress at a later stage in development (pollen mitosis) showed more severe effects in the male reproductive organs in Optic than in the other two varieties, with no viable pollen and significantly more sterility. The female reproductive organs of the three cultivars showed no severe effects after heat stress, at least in terms of embryo sac expansion. Overall, RGT Planet performed worst compared to the control conditions, whilst Moonshine performed best. However, there were less florets per spike in the heat stressed plants across all three varieties compared to control plants. Further investigation will be required to identify the traits that contribute to Moonshine being more heat tolerant than the other two varieties. The data provided here provides a basis for further studies into differential heat stress responses during key stages of barley reproductive development. Moreover, the use of X-ray imaging is a promising new method to visualise the morphology of floret organs without dissection and manipulation with chemicals, giving a more representative image and accurate volume measurements. Further work is needed to determine how the impact of the heat stress observed here equates to that under field conditions and the resultant potential consequences for yield loss. Nevertheless, significant yield reductions have been observed (Jacott and Boden, 2020), so it seems likely that equivalent impacts will be seen. The lines used here our elite cultivars that have not been particularly developed for heat stress resilience. However, the impact of the heat stress observed here can provide a basis for trait targeting for increasing future crop resilience.

## Data availability statement

The original contributions presented in the study are included in the article/**Supplementary Material**. Further inquiries can be directed to the corresponding author.

## Author contributions

CC, ZW, and DZ conceived the study. CC carried out the majority of experiments and data analysis. JFG assisted with plant maintenance and spike analysis. MT assisted with immunolabelling. All authors contributed to the article and approved the submitted version.

## Supplementary material

Supplementary Figure 1 Schematic of heat stress treatments during pollen development for barley varieties and pollen viability in barley cultivars Optic, Moonshine and RGT Planet. **(A)** Two pots with four plants each of the three varieties were submitted to either meiosis heat stress during meiosis I and II of pollen development, or mitosis heat stress during mitosis I and II of pollen development. **(B)** Pollen viability is shown from a randomly selected floret right before anthesis from heat stressed plants and control plants. Black pollen is viable, while yellow/brown pollen is not viable; low numbers of pollen reflect the lack of pollen in the mature anthers.

Supplementary Figure 2 Phylogenetic analysis of rice, Arabidopsis and barley HSP17.8 **(A)** and HSP70 orthologs **(B)**. The alignments were constructed using the Geneious alignment method and the Blosum62 cost matrix in Geneious. **(C D)** Phylogenetic tree of Arabidopsis BLAST analysis HSP17.8 **(C)** and HSP70 **(D)**.

Supplementary Figure 3 X-ray CT images of anther and carpel development in control and heat stress conditions for Moonshine. X-ray images of the anthers and carpels obtained by X-ray CT are compared for control environment and heat stress environment (heat stress during meiosis of pollen development) right before anthesis.

Supplementary Figure 4 Immunolabelling of cell wall components in anthers and ovules in Optic. Comparison of control and heat stressed anthers and ovules in barley florets of the variety RGT Planet. Antibodies include BG1 (1,3;1,4-β-glucan, green) and LM19 (low methylesterified pectin, red). Calcofluor White counterstain was used to detect cellulose and mixed-linkage glucan (blue).

Supplementary Figure 5 Immunolabelling of cell wall components in anthers and ovules in Moonshine. Comparison of control and heat stressed anthers and ovules in barley florets of the variety RGT Planet. Antibodies include BG1 (1,3;1,4-β-glucan, green) and LM19 (low methylesterified pectin, red). Calcofluor White counterstain was used to detect cellulose and mixed-linkage glucan (blue).

Supplementary Figure 6 Immunolabelling of cell wall components in anthers and ovules in RGT Planet. Comparison of control and heat stressed anthers and ovules in barley florets of the variety RGT Planet. Antibodies include BG1 (1,3;1,4-β-glucan, green) and LM19 (low methylesterified pectin, red). Calcofluor White counterstain was used to detect cellulose and mixed-linkage glucan (blue).
