# Supplementary material for: Heat stress responses vary during floret development in European spring barley cultivars
Source: Front Plant Sci. 2023 Feb 3;13:918730. doi: 10.3389/fpls.2022.918730 (PMC9936242; doi:10.3389/fpls.2022.918730)
Supplement: Supplementary Figure 1 — Schematic of heat stress treatments during pollen development for barley varieties and pollen viability in barley cultivars Optic, Moonshine and RGT Planet. (A) Two pots with four plants each of the three varieties were submitted to either meiosis heat stress during meiosis I and II of pollen development, or mitosis heat stress during mitosis I and II of pollen development. (B) Pollen viability is shown from a randomly selected floret right before anthesis from heat stressed plants and control plants. Black pollen is viable, while yellow/brown pollen is not viable; low numbers of pollen reflect the lack of pollen in the mature anthers. [file Image_1.pdf]

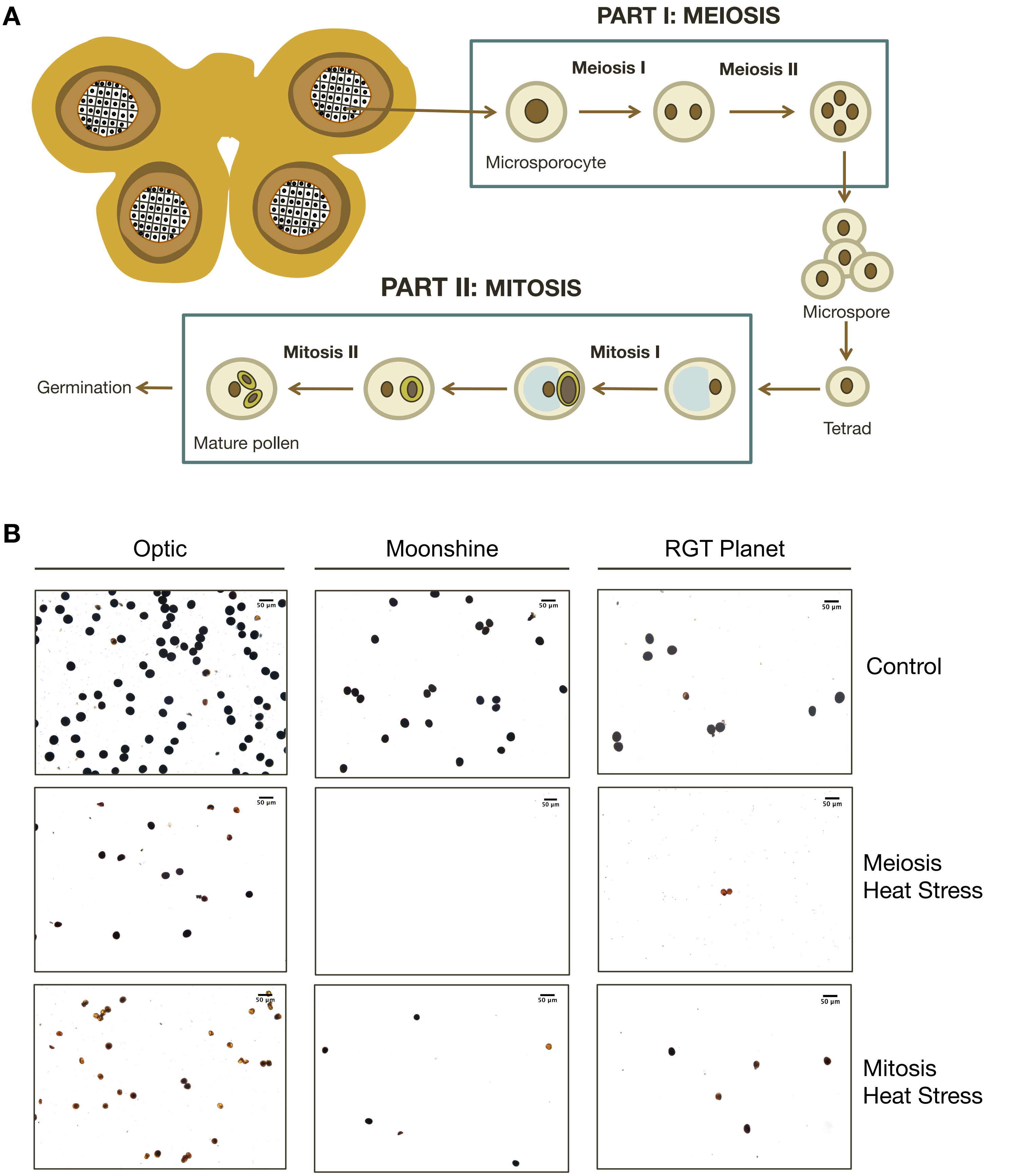

Supplementary Figure S1 Schematic of heat stress treatments during pollen development for barley varieties and pollen viability in barley cultivars Optic, Moonshine and RGT Planet. (A) Two pots with four plants each of the three varieties were submitted to either meiosis heat stress during meiosis I and II of pollen development, or mitosis heat stress during mitosis I and II of pollen development. (B) Pollen viability is shown from a randomly selected floret right before anthesis from heat stressed plants and control plants. Black pollen is viable, while yellow/brown pollen is not viable; low numbers of pollen reflect the lack of pollen in the mature anthers.

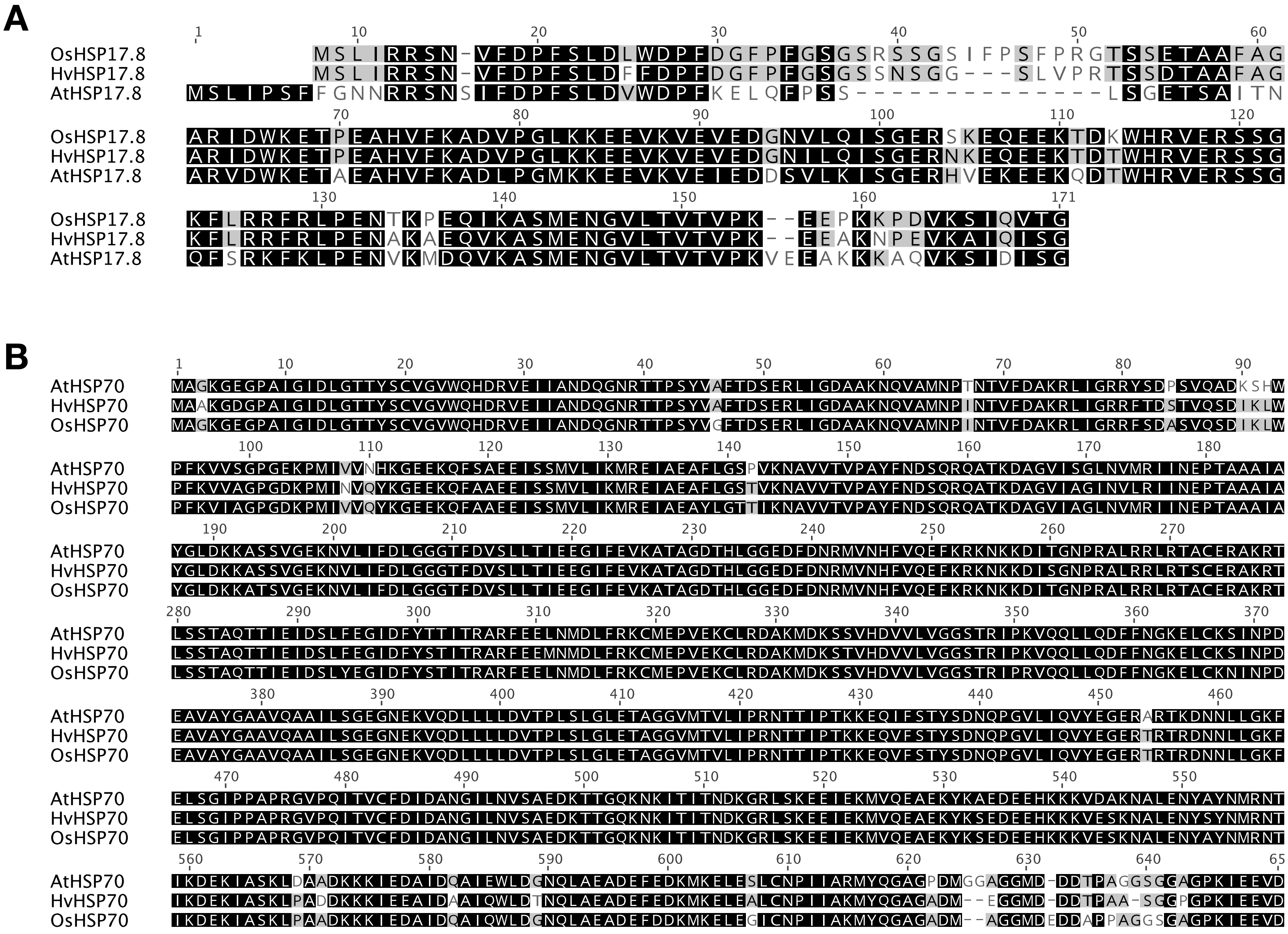

Supplementary Figure S2 Phylogenetic analysis of rice, Arabidopsis and barley HSP17.8 (A) and HSP70 orthologs (B). The alignments were constructed using the Geneious alignment method and the Blosum62 cost matrix in Geneious. **(C & D) Phylogenetic tree of Arabidopsis BLAST analysis HSP17.8 (C) and HSP70 (D).**

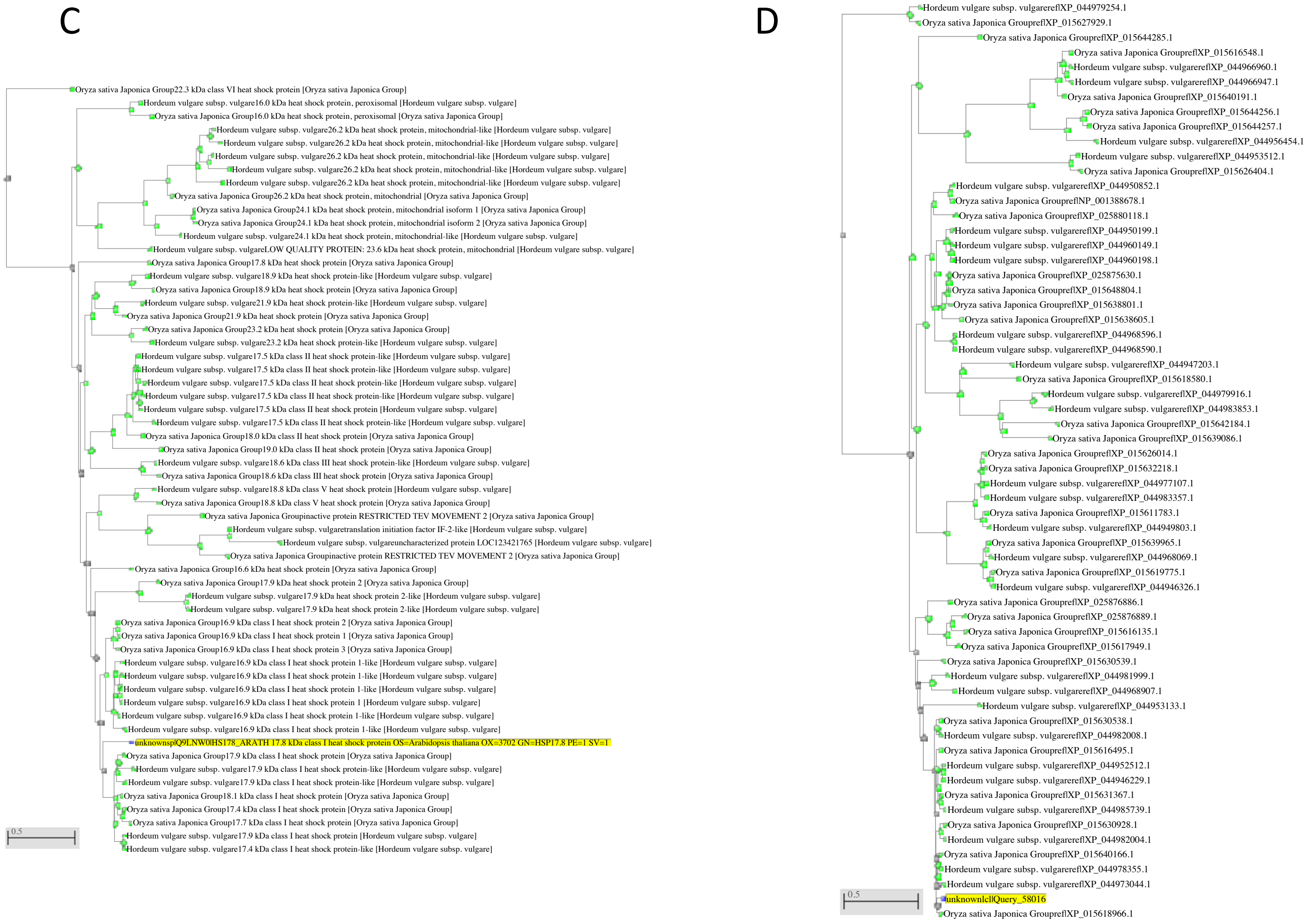

Supplementary Figure S2 Phylogenetic analysis of rice, Arabidopsis and barley HSP17.8 (A) and HSP70 orthologs (B). The alignments were constructed using the Geneious alignment method and the Blosum62 cost matrix in Geneious. **(C & D) Phylogenetic tree of Arabidopsis BLAST analysis HSP17.8 (C) and HSP70 (D).**

CONTROL MEIOSIS

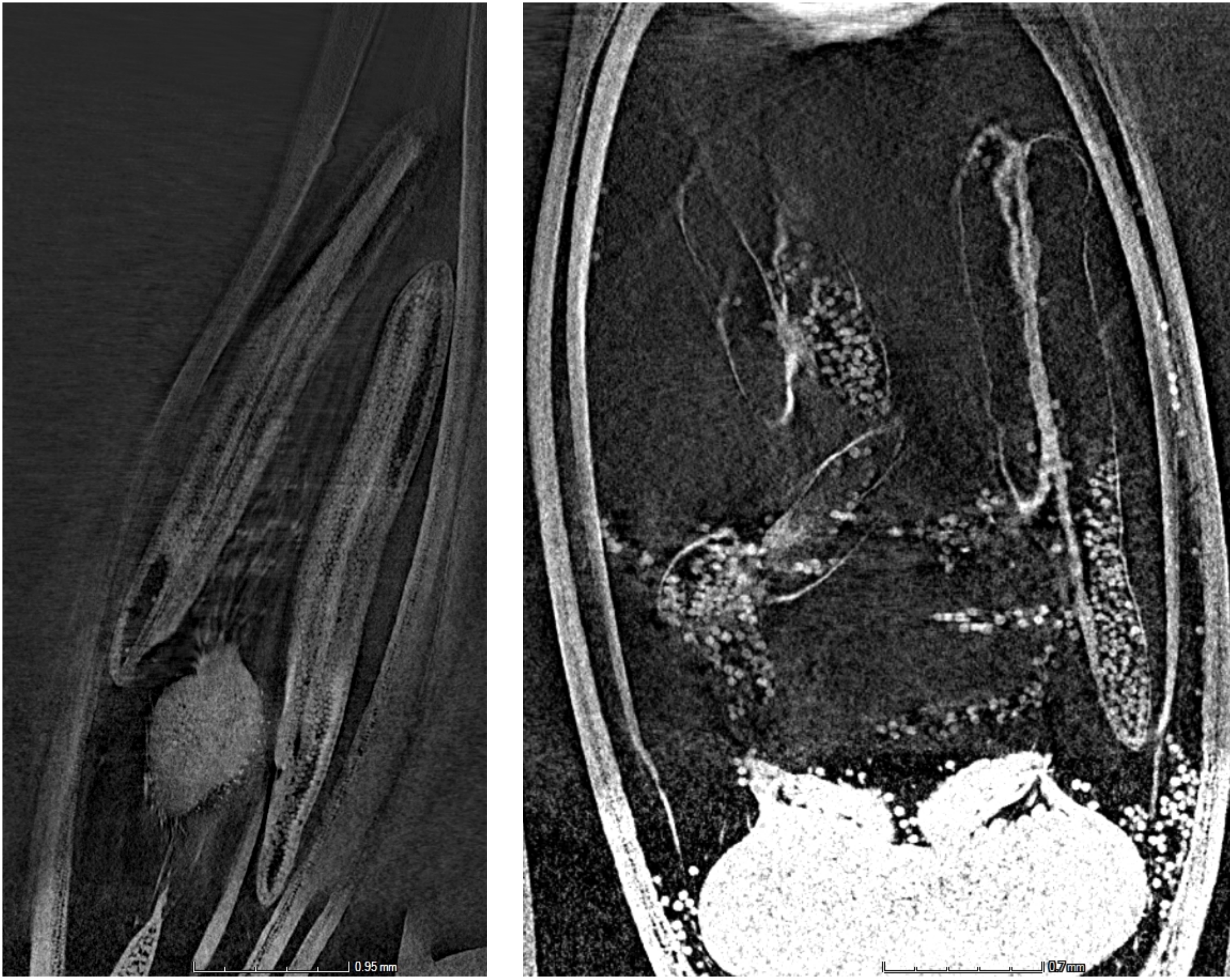

Supplementary Figure S3 X-ray CT images of anther and carpel development in control and heat stress conditions for Moonshine. X-ray images of the anthers and carpels obtained by X-ray CT are compared for control environment and heat stress environment (heat stress during meiosis of pollen development) right before anthesis.

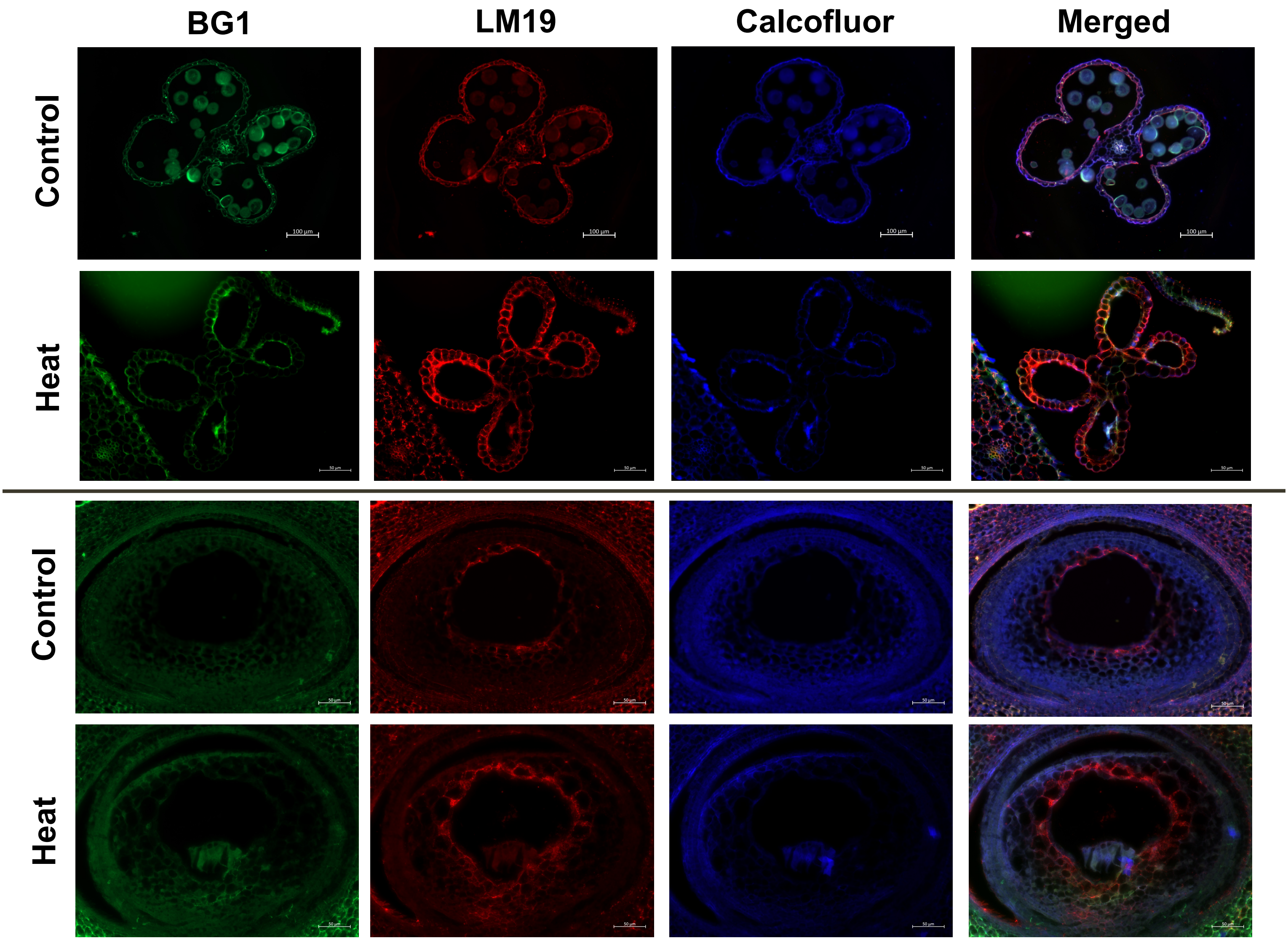

Supplementary Figure S4 Immunolabelling of cell wall components in anthers and ovules in Optic. Comparison of control and heat stressed anthers and ovules in barley florets of the variety RGT Planet. Antibodies include BG1 (1,3;1,4-β-glucan, green) and LM19 (low methylesterified pectin, red). Calcofluor White counterstain was used to detect cellulose and mixed-linkage glucan (blue).

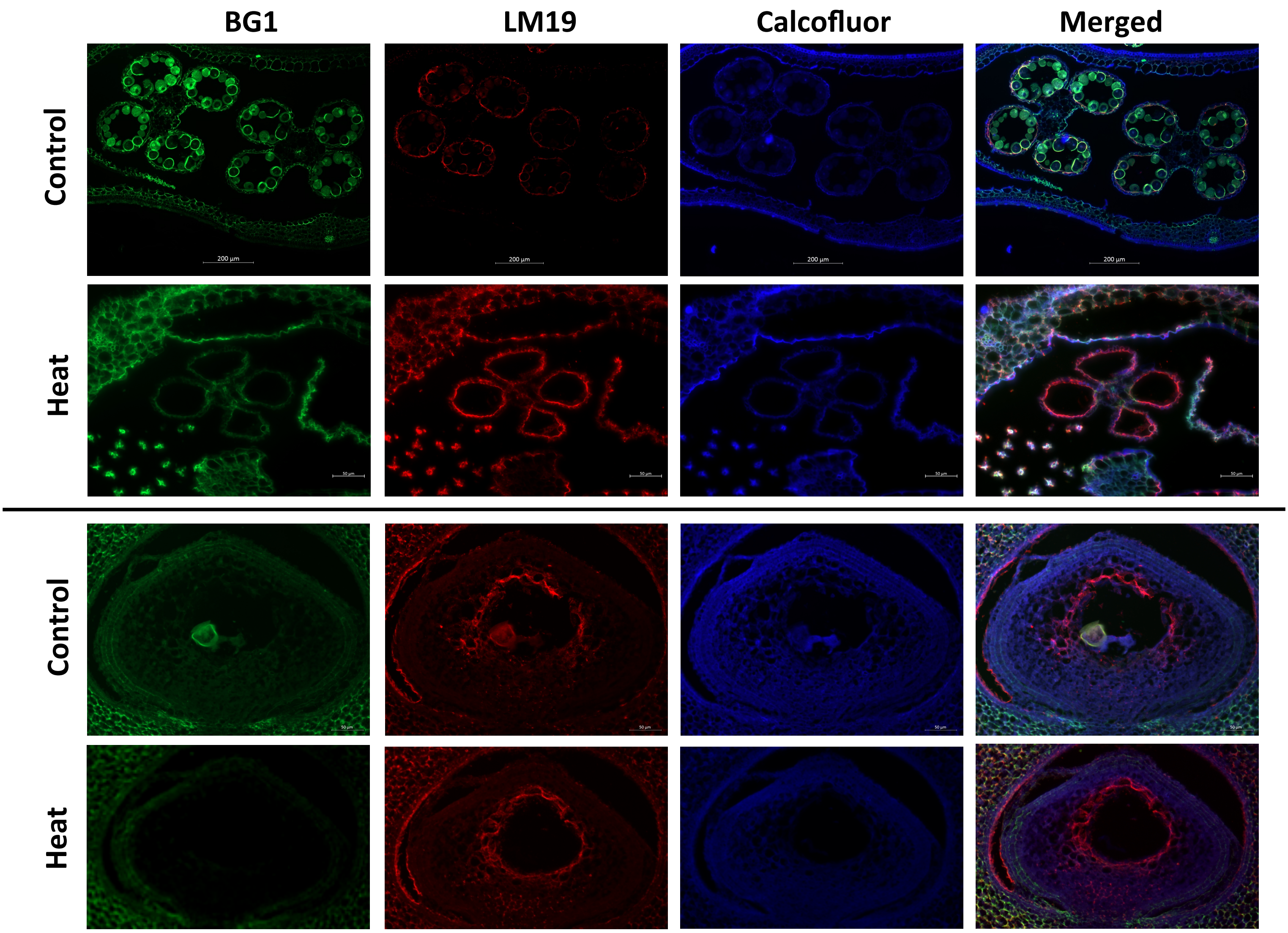

Supplementary Figure S5 Immunolabelling of cell wall components in anthers and ovules in Moonshine. Comparison of control and heat stressed anthers and ovules in barley florets of the variety RGT Planet. Antibodies include BG1 (1,3;1,4-β-glucan, green) and LM19 (low methylesterified pectin, red). Calcofluor White counterstain was used to detect cellulose and mixed-linkage glucan (blue).

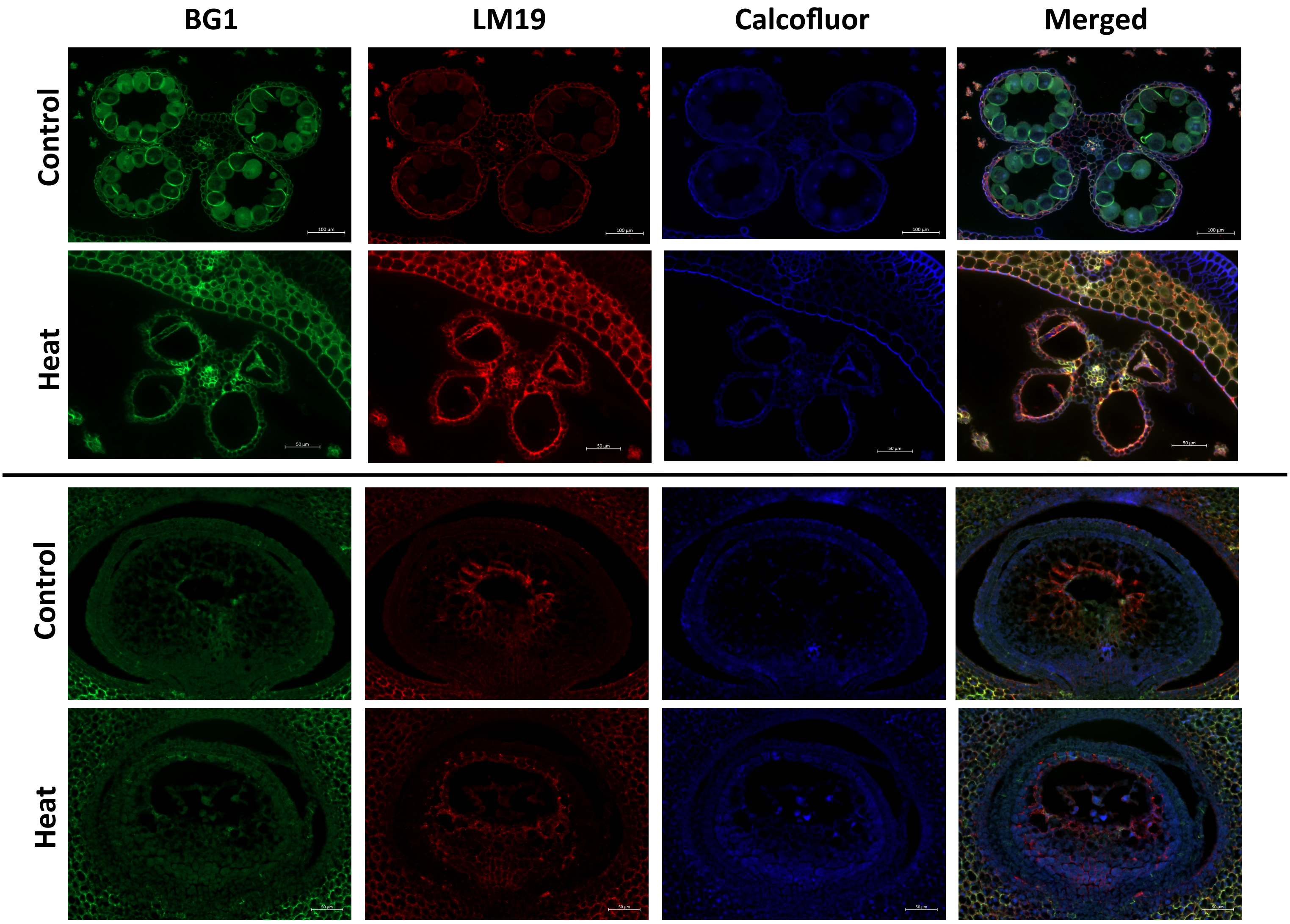

Supplementary Figure S6 Immunolabelling of cell wall components in anthers and ovules in RGT Planet. Comparison of control and heat stressed anthers and ovules in barley florets of the variety RGT Planet. Antibodies include BG1 (1,3;1,4-β-glucan, green) and LM19 (low methylesterified pectin, red). Calcofluor White counterstain was used to detect cellulose and mixed-linkage glucan (blue).
